# A comparison of clinical outcomes among people living with HIV of different age groups attending queen Elizabeth central hospital outpatient ART Clinic in Malawi

**DOI:** 10.3389/fmed.2023.1175553

**Published:** 2023-09-19

**Authors:** Jessica Chakakala-Chaziya, Noel Patson, Vincent Samuel, John Mbotwa, Danilo Buonsenso, Master Chisale, Eddie Phiri, Bernadette O’Hare

**Affiliations:** ^1^Department of Paediatrics and Child Health, Mzuzu Central Hospital, Ministry of Health, Lilongwe, Malawi; ^2^The School of Public Health, University of Witswaterands, Johannesburg, South Africa; ^3^The School of Public Health and Family Medicine, College of Medicine, University of Malawi, Zomba, Malawi; ^4^Malawi Liverpool Wellcome Trust Research Programme, Blantyre, Malawi; ^5^The Department of Applied Studies, Division of Statistics, Malawi University of Science and Technology, Mikolongwe, Malawi; ^6^Department of Woman and Child Health and Public Health, Fondazione Policlinico Universitario A. Gemelli IRCCS, Rome, Italy; ^7^Faculty of Science Technology and Innovations, Biological Sciences Department, Mzuzu University, Mzuzu, Malawi; ^8^The Umodzi Family ART Clinic, Queen Elizabeth Central Hospital, Blantyre, Malawi; ^9^Department of Paediatrics and Child Health, College of Medicine, University of Malawi, Zomba, Malawi; ^10^The Division of Infection and Global Health, University of St Andrews, St Andrews, United Kingdom

**Keywords:** virological suppression, adolescent HIV, pediatrics, retention, HIV continuum care

## Abstract

**Introduction:**

Adherence to Antiretroviral Treatment (ART) in children and adolescents living with HIV in low-resource settings is not extensively studied in large cohort studies including both adults and pediatric patients. We compared rates of virological suppression, adherence and defaulting among children, adolescents and adults attending a family ART clinic at Queen Elizabeth Central Hospital; a tertiary hospital situated in the southern region of Malawi.

**Methods:**

The study was longitudinal and made use of routinely collected data for all 27,229 clinic attendees. Clinical information obtained at routine clinical visits entered electronically since 2008 was extracted in February 2017. This data was used to ascertain differences across the different age groups. Logistic regression and Cox regression models were fitted to compare rates of Virological Suppression (VS), adherence, and defaulting, respectively.

**Results:**

Younger and older adolescents (ages 10–14 years and 15–19 years respectively) were less likely to achieve VS compared to adults in the final model AOR 0.4 (0.2–0.9, 95% CI) and AOR 0.2 (0.1–0.4, 95% CI) respectively. Young children (ages 0–4 years), older children (ages 5–9 years) and younger adolescents were less adherent to ART compared to adults AOR 0.1 (0.1–0.2, 95% CI), AOR 0.2 (0.1–0.3, 95% CI), and AOR 0.4 (0.3–0.5, 95% CI) respectively. Young adults and younger children had an increased likelihood of defaulting compared to adults.

**Conclusion:**

Poor performance on ART of children and adolescents highlights unaddressed challenges to adherence. Ongoing research to explore these potential barriers and possible interventions needs to be carried out. The adherence assessment methods used and strategies for improving it among children and adolescents need to be revised at the clinic.

## Introduction

The public health approach to HIV care is a cost effective method of achieving access to care for all people of all age groups living with HIV/AIDS (1). Its success results in high numbers being retained in care and achieving Virological Suppression (VS). This ultimately reduces AIDS associated morbidity and mortality, as well as community transmission (1).

The results of older studies in the area have demonstrated that with this standardized approach to care, children, and adolescents perform poorer compared to adults (2–6). Reasons given for this relatively poor performance are multifactorial and prominent among them are challenges to adherence to Antiretroviral Therapy (ART) in these younger age groups (4, 7, 8). The result has been an increment in research aimed at establishing factors influencing adherence and virological performance specific to these younger age groups.

Malawi adopted the public health approach to HIV care in 2004, when a decision to upscale ART was made by the Ministry of Health. Since then, as of 2015, approximately 1,091,656 people had been registered on ART (9). The rates of virological suppression among Malawian adults have been established (10). A 2012 study carried out a multicenter analysis of all people aged 15 years and older starting on ART at the country’s 3 central hospitals, namely Queen Elizabeth Central Hospital (QECH), Kamuzu Central Hospital (KCH), and Mzuzu Central Hospital (MCH) and one district hospital in Thyolo, found rates of VS by site to be 70, 84, 60, and 67%, respectively. They found an overall VS rate of 72% (10). Although these findings were below the target third 95 of the UNAIDS Fast-Track targets of 95-95-95, the most recent national estimates as of 2019 shows that the country is on track as the proportions of those with HIV ascertained, those who were HIV infected and on ART and clients who were virologically suppressed were 93.84 and 92%, respectively, National AIDS Commission (NAC) (11). Unfortunately, Wadondo et al. excluded younger adolescents and children hence could not demonstrate the care across all the populations age groups. Despite a rigorous search, there is no published data from the Malawian setting where a comparison of how adolescents and children being cared for using this public health approach fare in comparison to adults.

## Methodology

### Study design

A retrospective longitudinal methodology was employed to compare rates of VS, adherence and defaulting among children, adolescents and adults attending a family ART clinic at QECH; a tertiary hospital situated in the southern region of Malawi. Apart from being cost effective, this study design was considered in order to achieve the set objectives which required large sample size followed up for a significant period of time.

### Study place and setting

The study was conducted at QECH family ART clinic. QECH is the largest public health facility in Malawi and is a referral center for patients in the Southern Region of the country, the region with the highest HIV prevalence in the country at 12.8% (12). The ART clinic at QECH was established as a fee-paying clinic in 2000, but as of 2004 ART and other services have been provided free-of-charge. ART initiation criteria has evolved since the clinic was opened and has been based on the current national protocol which are adapted from the most recent WHO guidelines. As of June 2016 Malawi adopted the test and treat strategy and all adults and children living with HIV are eligible for ART regardless of immunological or clinical staging (13).

Besides ART, the clinic provides pre-ART and ongoing adherence counseling, routine clinical reviews for patients, Co-trimoxazole prophylaxis and recently Isoniazid prophylaxis. According to the algorithm for laboratory monitoring in the national guidelines, viral loads testing is done routinely, i.e., 6 months after ART initiation, then annually after starting on ART as of a recent 2019 Malawi addendum to clinical guidelines (14). Targeted viral loads are done on patients developing a World Health Organization (WHO) stage 3 or 4 illness who have been on ART for a minimum of 1 year (15). However, the algorithm is not always adhered to as a result of missing or long turnaround time of viral load results and poor implementation of testing protocols.

All other care is uniform across patients of different ages except for peer support groups such as teen clubs. Blantyre teen club was started in 2010 and evolved with the initial support of the Baylor International Pediatric Aids Initiative and consists of a network of peer support groups that aims to meet the specific medical and psychosocial needs of Adolescents living with HIV (ALHIV) to increase their rate of retention in care. The initiative is among many things reported to normalize the lives of ALHIV and improve adherence to medication. However, reports of its benefits have been anecdotal but more recent evidence is demonstrating that this form of supportive care decreases attrition rates (16, 17). The QECH teen club is scheduled once monthly however, the COVID-19 pandemic had resulted in interruptions to this schedule whose effects remain to be determined. The teen club clinic comprises of a morning clinic in which adolescents are reviewed and collect their ARTs followed by adolescent focused recreational activities. While most adolescent patients attending QECH ART clinic have been invited to attend the teen clinic and club, not all patronize the club. As of 2017 they were a total of 405 adolescents aged 12–19 years enrolled in the program, each monthly event was attended by approximately 140 adolescents.

### Study population and sampling methods

We used a convenient method of sampling to include data for all children, adult and adolescent patients attending QECH outpatient ART clinic for whom the clinical variables were available in the database to include in the analysis.

### Data management

Clinical and socio-demographic details of all ART initiators are routinely entered into the clinic’s electronic data management system at registration and at each subsequent visit since 2008. These records are backed up with paper records. However, data entry in the patient’s paper files on routine clinic visits is generally poor. There were 27,229 patients registered in the system. Data was available on demographic and clinical characteristics on all registered patients including the following demographic and clinical fields, in Table 1. Data was extracted from the system on the 22nd of February 2017 and edited on the 5th of June 2021 to include outstanding viral load results and then entered in Excel. It was then cleaned and reviewed.

**Table 1:** Demographic and clinical characteristics of 27,229 patients living with HIV on ART at QECH Blantyre, Malawi, stratified by age at ART enrollment.

	Young child 0–4 Years *N* = 2,125 (7.81%)	Older children 5–9 Years *N* = 1,130 (4.15%)	Young adolescents 10–14 Years *N* = 997 (3.66%)	Older adolescents 15–19 Years *N* = 672 (2.47%)	Young adult 20–24 Years *N* = 1,844 (6.78%)	Adults ≥ 25 years *N* = 20,442 (75.13%)	Total *N* = 27,229
Gender male	1,065 (50.12%)	629 (55.66%)	472 (47.34%)	230 (34.23%)	363 (19.69%)	9,120 (44.61%)	11, 879 (43.6)
Proportion of total with viral load result	770 (36.2%)	591 (52.3%)	418 (41.9%)	258 (38.39%)	579 (31.3%)	8,582 (41.9%)	11, 198 (41%)
Detectable viral load	301 (39.09%)	228 (38.58%)	197 (47.13%)	97 (37.60%)	112 (19.34%)	1,305 (15.21%)	2,240 (20%)
Poor adherence	597 (87.92%)	226 (81.29%)	205 (70.93%)	115 (58.67%)	340 (52.55%)	3,287 (53.79%)	4,770 (58.17%)
Treatment line on, 1st Line	1919 (90.3%)	927 (82.04%)	851 (85.36%)	590 (87.8%)	1768 (95.88%)	19,307 (94.4%)	25,362 (93.2%)
Treatment line on 2nd Line	206 (9.69%)	203 (17.9%)	146 (14.6%)	82 (12.20%)	76 (4.12%)	1,135 (5.5%)	1,848 (6.79%)
Art duration less than 1 year	927 (43.62%)	262 (23.21%)	275 (27.58%)	247 (36.76%)	688 (37.31%)	5,877 (28.75%)	8,276 (30.42%)
Art duration more than 1 year	1,198 (56.38%)	867 (76.79%)	722 (72.42%)	425 (63.24%)	1,156 (62.69%)	14,563 (71.25%)	18,931 (69.58%)
*WHO stage at ART initiation*							
WHO I and II	689 (32.42%)	300 (26.55%)	304 (30.49%)	266 (39.58%)	849 (46.04%)	8,540 (41.78%)	10,948 (40.24%)
WHO III and IV	1,436 (67.58%)	830 (73.45%)	693 (69.51%)	406 (60.42%)	995 (53.96%)	11,902 (58.22%)	16,262 (59.76%)
*TB status infection*							
Confirmed infection	43 (2.41%)	24 (2.50%)	23 (2.76%)	19 (3.36%)	57 (3.80%)	588 (3.49%)	754 (3.35%)
*Outcome status*							
Defaulter	759 (36.60%)	238 (21.58%)	255 (26.62%)	209 (32.20%)	626 (35.33%)	5,302 (26.60%)	7,389 (27.89%)
Died	74 (3.57%)	30 (2.72%)	43 (4.49%)	43 (6.63%)	66 (3.72%)	1,198 (6.01%)	1,454 (5.49)
Alive On ART	757 (36.50%)	572 (51.86%)	437 (45.62%)	268 (41.29%)	647 (36.51%)	9,481 (47.56%)	12,162 (45.91%)
Stopped	7 (0.34%)	1 (0.09%)	0	2 (0.31%)	5 (0.28%)	49 (0.25%)	64 (0.24%)
Transferred out	477 (23%)	262 (23.75%)	223 (23.28%)	127 (19.57%)	428 (24.15%)	3,904 (19.58%)	5,421 (20.46%)
Rate of retention to care	47.5%	68.01%	59.4%	51.3%	48.1%	59.1%	57.7%
Switch to alternate first line	1,122 (52.80%)	752 (66.55%)	556 (55.77%)	301 (44.79%)	825 (44.74%)	11,309 (55.32%)	14,865 (54.63%)

### Definition and outcomes

#### Age categorization

We used the age at ART enrolment to categorize the patients by age. Children (0–9 years) were classified into young children (0–4 years) and older children (5–9 years). Adolescents (10–19 years) were also further classified into young adolescents ages (10–14 years) and older adolescents (15–19 years). Young adults were those aged 20–24 years and adults as those aged 25 years and above. This categorization was used to facilitate comparison in performance on ART of these age groups with findings in other studies conducted in the African region and other parts of the world.

#### Virological suppression

This was classified into (VS) which was defined as the most recent routine or targeted viral load of less than 1,000 RNA copies/μL or Virological Non-Suppression (VNS) the most recent routine or targeted viral load of 1,000 RNA copies/μL or above.

The independent variables for the overall analysis included:

#### Who clinical stage

This was categorized into WHO stage 1 and 2 or stage 3 and 4.

#### TB co-infection status

Confirmed TB was defined by a clinician-driven diagnosis of TB, or non-TB if the patient was not suspected of having TB.

#### ART regimen type

The regimen types were grouped into Nevirapine, Efavirenz and Protease Inhibitor based and Non-standard Regimen. At the time of preparing this manuscript, our center used a combination of lamivudine and zidovudine, Lamivudine and stavudine, Lamivudine and abacavir, together with an nevirapine, efavirenz, or a protease inhibitor. At the time integrase inhibitors had not been introduced to Malawi’s public health approach ART combinations.

#### Adherence status

Adherence was classified as good if the most recent adherence as determined by an automated electronic system that utilizes an objective pill count was between 95 and 105% and poor if the same was outside this range

#### Clinical outcome

We used the following outcome definition.

Retained to care: any person known to be alive on ART at the time of data extraction.

*Defaulter*: any person who was known to be on ART and had not returned for follow up for 2 months and was not known to have transferred out, stopped ART or died.

*Died*: the patient was known to have died.

*Stopped*: the patient was last known to be alive and had stopped taking ART whether because of a personal or a clinician’s decision.

*Transfer out*: the patient was known to be alive and on ART but had been transferred out to another ART clinic.

*Alive on ART*: was known to be alive and receiving ART at the QECH family clinic.

### Statistical analysis

Descriptive analyses were used to stratify patients clinical and demographic characteristics by virological status and to calculate rates of defaulting, retention to care by different age category in the univariate analyses, the Pearson Chi-square test was used to compare the virological status and other categorical variables. A logistic regression model was fitted to determine factors associated with the VS. All factors found to be significant at a value of *p* of less than 0.2 in the bivariate logistic model were included in the final model that made use of a backward stepwise approach of elimination. In the final model, all factors with *p*-values less than 0.05 were taken to be significant. A survival analysis was used to calculate the overall rate of defaulting and the rates were also calculated by age category. Kaplan Meier curves were plotted with defaulting as the outcome of interest and log-rank tests to compare survival times by age category were also computed. A Cox regression model was fitted to identify factors associated with defaulting. All the analyses were done using StataCorp. 2013. Stata Statistical software: Release 13. College Station, TX: StataCorp LP and windows Excel version.

#### Ethical considerations

Ethics approval to conduct this study was obtained from the College of Medicine Research and Ethics approval board protocol P.10/16/2050. To ascertain patient confidentiality clinic numbers were used to identify all study participants. Consent to utilize the data was obtain from facility pediatric department, ART clinic and Hospital Management. Being retrospective study that made use of routinely collected clinic data, no consent was obtained from the individual participants.

## Results

### Demographic and clinical characteristics

The QECH ART clinic database had 27,229 ART patients who had ever been registered, of whom 56% were female and 44% were male. The young adult age category had the largest disparity among genders with 80% being female. Adult population (75%), young adults (ages 20–24 years) 7%, and with the sub-classifications combined, children (0–9 years) made up 12% and adolescents (10–19 years) 6%. See Table 1.

**Table 2:** Factors associated with VS (1,000 RNA copies/μL) in patients on ART^a^.

	Undetectable *n* (%)	Odds ratio 95% CI	*p*-value	Adjusted odds ratio 95% CI^b^	*p*-value
*Gender (total n)* Male (4,584) Females (6,620)	3,533 (77%) 5,427 (82%)	1 1.3 (1.2–1.4)	<0.001		
*Duration on ART* 1 year to less than 1 year (648) More than 1 year-10 years (8,417) more than 10 years-19 years (2,139)	472 (73%) 6,782 (80%) 1,706 (80%)	1 1.5 (1.2–1.8) 1.4 (1.1–1.7)	<0.001 <0.001		
*Age at ART initiation* Adult (8,582) young adult (579) older teen (258) younger teen (418) older child (591) young child (770)^a^	7,277 (84%) 467 (82%) 161 (58%) 221 (53%) 363 (64%) 469 (60%)	1 0.7 (0.6–0.9) 0.3 (0.2–0.4) 0.2 (0.1–0.2) 0.3 (0.2–0.3) 0.3 (0.2–0.3)	0.008 <0.001 <0.001 <0.001 <0.001	0.8 (0.5–1.4) 0.3 (0.1–0.9) 0.2 (0.1–0.4) 1.0 (0.4–2.4) 0.6 (0.3–1.06)	0.508 0.029 <0.001 0.988 0.071
*TB co-infection status* Confirmed TB (117) No TB (10,853)	8,727 (80%) 60 (51%)	–1 3.8 (2.7–5.6)	<0.001		
*Defaulting* *^c^* Not Defaulters (9,849) Defaulters (1,294)	7,899 (82%) 856 (66%)	–1 0.4 (0.3–0.4)	< 0.001	0.7 (0.5–1.02)	0.076
*WHO stage* *^d^* *at initiation* WHO stage 1 & 2 (4,808) WHO stage 3 or 4 (6,396)	3,933 (82%) 5,027 (79%)	1 0.8 (0.7–0 0.8)	<0.001		
*Adherence* Poor adherence (468) Good adherence (456)	329 (70%) 351 (77%)	1.4 (1.05–1.8)	0.022	1.3 (0.9–1.8)	0.068
*Regimen dosage* Once daily (4,909) Twice daily (6,248)	4,350 (89%) 4,576 (73%)	0.3 (0.3–0.4)	<0.001		

a Analysis only included 11,204 patients with viral load result available.

b Adjusted for factors with value of *p* < 0.2 in bivariate analysis Tb class, defaulter status, who stage at ART initiation, age category at ART initiation duration on ART, Gender, Frequency of ART regimen on. Adherence was retained for its clinical significance.

c Deaths removed from outcomes in the analysis.

d World Health Organization clinical staging for HIV and AIDS.

The first patient started ART in 1999, and the most recent start date in the data analyzed was February 2017. Most of the patients, regardless of age category, had been on ART for between 1 and 10 years. The longest duration on ART was 18.35 years, and this was a 35-year-old man who initiated ART as an older adolescent. He had initiated ART at the age of 17 and, as of the data extraction date, was on a first-line regimen of a fixed drug combination of Zidovudine/Lamivudine/Nevirapine).

At ART initiation, 40% were in WHO clinical stages 1 or 2, 60% in WHO stage 3, and 15% in WHO stage 4. The ART eligibility criteria for young children (0–5 years) evolved over the time period under review in line with national guidelines. The main indications for ART eligibility criteria in this age group were advanced WHO stages, i.e., WHO stages 3 and 4, (69%), followed by age-specific immunological cut-off (12%) as the second most common cause, and then by presumed severe HIV disease (PSHD) [9%; see Supplementary information Table 1; where access to virological testing is not routinely available, a presumptive diagnosis of severe HIV (PSHD)] disease can be made in infants and children who are less than 18 months of age with a positive serological HIV test (in either the mother or child) and who have specific symptoms suggestive of HIV infection, according to the WHO and national guidelines (18).

The proportion of those starting ART at an advanced clinical stage, WHO stage 3 and 4, was highest among younger adolescents (74%), older adolescents (69%) and older children (68%) compared to the other age divisions. At the time of data extraction 3% had confirmed TB and 80% of these were on treatment (see Table 2). The proportion of TB cases among children was like adults. Both young and older adolescents had the smallest proportion of confirmed TB cases.

Among all the clinic attendees, 93% were on first line treatment, of these 55% had been switched to an alternate first line. Only 7% were on second line. Regarding ART regime, 35% were on a Nevirapine based regime, 35% on an Efavirenz based regime, and 5% on a Protease Inhibitor based regime and 25% on a non-standard regime.

### Comparison of performance across the age groups

#### Virological suppression and adherence performance

Eleven thousand nine hundred and seventy-nine (41%) of the 27,229 ART patients had a viral load result available in the system. The overall clinic VS rate was 80%. Adults had the highest rate of VS at 85% and young adolescents had the lowest rate at 53% (see Table 2). Even though at the bivariate analysis all younger age categories had a reduced odds of VS, in the final model of the logistic regression only older and younger adolescents where found to have significantly reduced likelihood of VS Adjusted Odds Ratio (AOR); 0.3 (0.1–0.9 95% CI); and 0.2 (0.1–0.4 95% CI) respectively (see Table 2).

Eight thousand two hundred (30%) of the all-clinic attendees had an adherence percentage indicated. The overall clinic adherence rate was 42%. The highest rate of good adherence percentage was 47% and this was among young adults. Good adherence rates decreased with age; young children experienced the lowest rate of good adherence of 12% (see Table 2). In the final model younger adolescents, older and younger children had a reduced likelihood of achieving good adherence compared to adults AOR 0.4 (0.3–0.5, 95% CI), 0.2 (0.1–0.3, 95% CI), and 0.1 (0.1–0.2, 95% CI) respectively (Supplementary Material 3).

#### Clinical outcomes

Since the use of the electronic record system in 2008 at the QECH family ART clinic, 12,162 (46%) of its patients were alive and on ART, 7,393 (28%) had defaulted, 5,421(21%) had transferred out to other clinics and 1,454 (6%) had died. The clinic overall rate of retention to care was 58%. Older children had the highest rate of retention to care at 68% young children and young adults had the lowest rate of retention to care of 48% and 48%, respectively (see Table 1). The median time to death and to defaulting among those who experienced these outcomes was 4.7 months, IQR (0.98–25.25) and 9.6 months, IQR (1.31–35.59), respectively.

**Figure 1:**
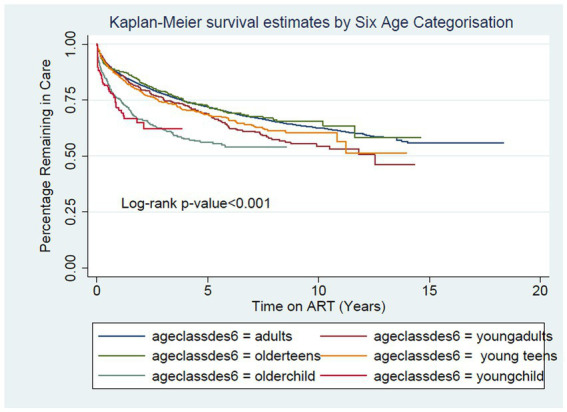
Crude Kaplan Curve showing percentage remaining in care by 6 age categorization [Adults (*n* = 22,006) Young Adults (*n* = 972) Older Adolescents (*n* = 978) Young Adolescents (*n* = 1,312) Older Child (*n* = 1,019) Young Children (*n* = 219)] when outcome of interest is defaulting.

The overall clinic default rate was 6.5/100pys. The default rates were statistically different crude Kaplan–Meier Curves, Log-rank *p* value <0.001 (see; young children and young adults experienced the highest default rate at 12.2/100pys and 10.1/100pys, respectively) (see Figure 1). Adults had the lowest rate at 6.02/100pys (see Supplementary Material 10). Those of older children, young adolescents and older adolescents were like the adult rate at 5.91/100pys, 6.6/100pys, and 9.5/100pys, respectively. In the Multivariable Cox regression models the relationship remained the same with young adults and young children had increased odds of defaulting AHR 1.3 (1.1–1.5 95% CI) and 1.5 (1.2–1.7 95% CI) respectively.

## Discussion

To our knowledge, this is the largest study comparing VS, adherence and clinical outcomes in large cohorts of pediatric and adult patients assessed in our setting. We found that adults had the highest rate of VS. All children and adolescent subsets performed poorer in comparison. However, in the multivariate analysis only adolescents were found to be significantly less likely to achieve VS compared to adults. All subsets of children and young adolescents were also less likely to achieve good adherence. Young adults and young children were more likely to default compared to adults.

The rate of VS among adults was 85%, while young and older adolescents on ART attending QECH were less likely to achieve virological suppression, in line with previous studies (2, 7). However one study looking at outcomes of South African adolescents on ART found the rates of VS at 6 months on ART among young and older adolescents to be higher at 86 and 84%, respectively (7). This South African study had a high number of participants missing viral loads (68%) which might have resulted to a bias.

The rates of VS among young and older children in this study were 61 and 61%, respectively. The rates of VS among children on ART in low resource settings have a wide variation, ranging from 29 to 87% (19–21). Our rates are lower than some have found in the region. A study in Tanzania and one in Botswana found rates of approximately 75 and 80%, respectively (22, 23). The excellent Tanzanian results may have been as a result of the availability of specialized health care workers (23). Likewise the Botswana study was also carried at a pediatric unit with an active tracing program for patients missing appointments (22). Our clinic is a family clinic and therefore not specialized, at the time the study was being conducted defaulter tracing was only available on a small scale for patients who miss their appointments.

The virological status reflected the adherence performance as age groups with highest rates of non-adherence also had the highest rates of VNS. The proportion of adults achieving good adherence was 46% and that of younger and older adolescents was 29 and 41%, respectively. The adherence rates of adults and younger adolescents reflect those found by other researchers who conducted their study in 9 southern African Countries who found rates of adherence in adults and adolescents to be 41 and 21%, respectively, Nachega et al. (4). However, the adherence rate of older adolescents in our study was similar to that of adults. As expected from the adherence rates, young and older children, and younger adolescents were found to be less likely to attain good adherence compared to adults. Others have also found adolescents to be at reduced odds of achieving good adherence (4). In our final model adherence was not significantly associated with virological status. We suspect this is because the computed adherences might at times be an overestimate as pills are dispensed based on the closest approximations from the number in a bottle and not exactly the amount required to cover the period before the next appointment.

The findings of median times to an adverse event, i.e., death and defaulting was 4.7 months and 9.6 months, respectively, for all clinic attendees. This was similar to what others have found in their clinics (7). Young children and young adults had the highest rates of defaulting and also increase odds of defaulting compared to adults. A study done on children and adolescents (less than 15 years) in rural Mozambique found a similar default rate of 39% (24). The high default rates among children in our clinic could be as a result the absence of a defaulter tracing program to bring back patients to care, or also to confirm them as still being alive as a study done in Malawi found that 50% of those initially labeled as defaulters had died upon tracing, 23% were still alive and among these a third had transferred themselves to another clinic without informing their initial hospital. Also among these 23% who were still alive, others had stopped coming to the clinic because they had no transport money (25). As also found by Nglazi et al. our cohort of adolescents were not an increased odds of defaulting compared to adults (5). A South African study found young adolescents less likely but older adolescents more likely to default compared to adults (26).

This study was done to establish how the different age groups who are provided care at QECH ART Clinic perform on ART.

The World Health Organization (WHO) proposed support groups as an intervention to promote retention and adherence among people living with HIV (PLHIV) receiving ART (27, 28). Both WHO and the President’s Emergency Plan for AIDS Relief (PEPFAR) indicated and encouraged peer support groups facilitated by trained PLHIV to address the special needs of fellow PLHIV and their partners as a model which can best help managing PLHIV in the world (27, 28). However, our study shows that, similarly to other studies, younger age groups on ART at Queen Elizabeth Central Hospital perform poorer than adults on ART (3, 5, 26, 29). As others have suggested these findings illustrate the challenges that the younger population on ART faces with regards to adherence to ART (2, 6, 30, 31), suggesting that new strategies need to be implemented. In our setting as are many it brings to question whether a public health approach of standardizing care leaves the child or the adolescent living with HIV underserved with regards to adequately addressing age-specific barriers to adherence. The evidence on the effects of adolescent focused care on health outcomes such as virological suppression and retention to care is conflicting. Research done locally by Mackenzie et al. showed lower attrition rates among those accessing adolescents specialized care and other regional studies demonstrated no difference in outcomes between those accessing adolescents specialized care compared to those receiving a center not specialized in adolescents’ care (17). Other regional research also illustrates that clinics with defaulter tracing programs had higher rates of virological suppression among children (22, 23). The local evidence on the role of adolescent focused care does support the implementation of teen clubs on an opt out basis for all adolescents who are aware of their HIV status with ongoing evaluation of content and mode of delivery reflecting upcoming best practices in this crucial area of HIV care.

## Conclusion

We found that children and adolescents receiving ART at QECH hospital perform poorer compared to adults across various performance indicators including adherence, default rate and VS. This clearly highlights unmet or inadequately addressed needs among these age groups with our current approach to care. Care aimed at addressing age-specific needs is proving to yield better outcomes and tools used in the clinic for assessing adherence need to be addressed.

## Data availability statement

The raw data supporting the conclusions of this article will be made available by the authors, without undue reservation.

## Ethics statement

The studies involving humans were approved by College of Medicine Research and Ethics approval board protocol P.10/16/2050. The studies were conducted in accordance with the local legislation and institutional requirements. Written informed consent for participation in this study was provided by the participants’ legal guardians/next of kin.

## Author contributions

JC-C, NP, VS, JM, MC, EP, and BO’H were responsible for patients management, data collection, analyses, writing, and supervision. DB contributed with writing, conceptualization, and submission procedures. All authors contributed to the article and approved the submitted version.

## Conflict of interest

The authors declare that the research was conducted in the absence of any commercial or financial relationships that could be construed as a potential conflict of interest.

## Publisher’s note

All claims expressed in this article are solely those of the authors and do not necessarily represent those of their affiliated organizations, or those of the publisher, the editors and the reviewers. Any product that may be evaluated in this article, or claim that may be made by its manufacturer, is not guaranteed or endorsed by the publisher.
