# Supplementary material for: A comparison of clinical outcomes among people living with HIV of different age groups attending queen Elizabeth central hospital outpatient ART Clinic in Malawi
Source: Front Med (Lausanne). 2023 Sep 19;10:1175553. doi: 10.3389/fmed.2023.1175553 (PMC10546013; doi:10.3389/fmed.2023.1175553)
Supplement: Supplementary file 1 [file Table_1.docx]

**Supporting Information;** **A comparison of clinical outcomes amongst people living with HIV of different age groups attending Queen Elizabeth Central Hospital Outpatient ART Clinic in Malawi**

**S1 Table. Criteria for ART eligibility amongst those enrolling as young children.**

| **Eligibility** | **N (%)** |
| --- | --- |
| Immunological cut-off | 257 (12.2) |
| DNA PCR infant | 54 (2.6) |
| HIV infected | 173 (8.2) |
| PSHD | 187 (8.9) |
| WHO stage | 1436 (68.2) |

**S2 Table. The duration on ART by age at ART enrolment.**

|  | **0-1 year** | **More than 1 year-10years** | **More than 10years-19years** |
| --- | --- | --- | --- |
| Adult | 5882 (27.78) | 12386(60.60) | 2172(10.63) |
| Young adult | 688 (37.31) | 1074(58.24) | 82(4.45) |
| Older teenager | 247 (36.76) | 388(57.74) | 37(5.57) |
| Younger teenager | 275 (27.58) | 654(65.60) | 68(6.82) |
| Older child | 262 (23.21) | 738(65.37) | 129(11.43) |
| Younger child | 928 (43.67) | 1093(57.44) | 104(4.89) |

**S3 Table. Factors associated with adherence at QECH ART clinic.**

|  | **Good Adherence n (%)** | **Odds ratio 95%CI** | **P-value** | **Adjusted^a^ OR**  **95% CI^a^** | P-value |
| --- | --- | --- | --- | --- | --- |
| **Gender (total n)**  Male (3673)  Females (4,531) | 1,526(41.55%)  1,904 (42.02%) | 1.0(0.9-1.1) | 0.664 |  |  |
| Age at ART Initiation  Adult (6,111)  young adult (647)  older teen (196)  younger teen (289)  older child (278)  young child (679) | 2,824 (46.2%)1  307 (47.45%)  81 (41.33)  84 (29.07%)  52 (18.71%)  82 (12.08%) | 1.0(0.9-1.2)  0.8(0.6-1.09)  0.5 (0.4-0.6) 0.3(0.2-0.3)  0.1(0.1-0.2) | 0.548  <0.177  <0.001  <0.001  <0.001 | 1.0(0.9-1.2)  0.8(0.6-1.1)  0.4(0.3-0.5)  0.2(0.1-0.3)  0.1(0.1-0.2) | 0.285  0.259  <0.001  <0.001  <0.001 |
| **TB Co-infection Status**  No TB (5181 )  Confirmed TB (304 ) | 2,570(49.60%)  143(47.04%) | 1.1(0.9-1.3) | 0.385 |  |  |
| **WHO stage^a^ at initiation**  WHO stage 1 & 2 (2,636) WHO stage 3 or 4 (5,568) | 1,156 (43.85%)  2,274(40.84%) | 1.4(1.3-1.4) | <0.001 |  |  |
| **Duration on ART**  1year to less than 1year **(3,846 )**  More than 1year-10years**(4,333**)  more than 10years-19years**(24)** | 1,332 (34.6%)  2,086(48.14 %)  12(50.00%) | 1.7(1.6-1.9)  1.8(0.8-4.2) | <0.001  0.121 | 1.7(1.5-1.7)  1.4(0.6-3.2) | <0.001  0.360 |
| **Switch to alternate line**  **No (5,162)**  **Yes (3,042)** | 2,006 (38.86)  1,424(46.81) | 1.2(1.2-1.3) | <0.001 | 1.4(1.3-1.5) | <0.001 |

^a^ Adjusted for all variables with p-value of less than 0.2 on bivariate analysis with the adherence level which were being switched to alternate first line, WHO stage at ART initiation and duration on ART

**S4 Table. Risk factors for defaulting amongst all clinic attendees.**

|  | **Defaulters n (%)** | **Hazard ratio (95%CI)** | **P-value** | **Adjusted HR**  **95% CI^a^** | **P-value** |
| --- | --- | --- | --- | --- | --- |
| Gender (total n)  Male (11,542)  Females (14,964) | 3475(30 %)  3918 (26%) | 0.8(0.7-0.85) | <0.001 | 0.8(0.7- 0.87) | <0.001 |
| Age at ART Initiation  Adult (19,934 )  young adult (1,772 )  older teen ( 649 )  younger teen (958)  older child (1,103)  young child (2,074) | 5,302 (27 %)  626 (35 %)  43 (6.63 %)  43(4.49%)  30 (2.72%)  74 (3.57%) | 1.5(1.4-1.7)  1.4(1.2-1.7)  1.0 (0.8-1.2)  0.9(0.8-1.09)  1.7(1.6-1.9) | <0.001  <0.001  0.658  0.451  <0.001 | 1.3(1.1-1.5)  1.1(1.1-1.4)  0.9(0.7-1.2)  0.7(0.6-1.01)  1.5(1.2-1.7) | <0.001  0.192  0.912  0.066  <0.001 |
| TB Co-infection Status  No TB (21,085 )  Confirmed TB (710) | 4,566 (22%)  324 (46%) | 4.7(4.0-5.5) | <0.001 | 1.7(1.4-2.1) | <0.001 |
| WHO stage^a^ at initiation  WHO stage 1 & 2 (10,488)  WHO stage 3 or 4 (16,018) | 335 (3.19%)  1,120(6.99%) | 1.4(1.3-1.4) | <0.001 |  |  |
| Adherence  Poor Adherence (4,774 )  Good Adherence (3,430) | 1,671(35%)  1,482 (43%) | 0.9(0 .8-0.98) | 0.020 | 0.9(0.8-0.9) | 0.002 |
| Regiment dose  Once daily(8,367)  Twice daily(17,079) | 1,286(15%)  5201(30%) | 3.5(3.2-3.7) | <0.001 |  |  |
| ART Group  Niverapine based(9,002)  Efavirenz based (8,4950  Protease Inhibitor based(1,313)  Non-standard (6,636) | 3,425 (38 %)  1,300 (15%)  162 (12%)  1,268(19 %) | 0.2(0 .18-0.2)  0.2( 0.17-0.25)  0.4( 0.4-0.52) | <0.001  <0.001  <0.001 | 0.7( 0.6-0.8)  0.4(0.3-0.6)  1.9(1.7-2.1) | <0.001  <0.001  <0.001 |
| ART Line  First Line(24,698 )  Second Line(1,808) | 7,151 (29%)  242 (13%) | 0.4(0.4-0.5) | <0.001 | 0.3(0.2-0.43) | <0.001 |

^a^ Adjusted for factors with p-value<0.2 in bivariate analysis TB class, age at ART initiation, Adherence, WHO stage at ART initiation, ART group, frequency of medication and Gender.

^b^ World Health Organization Clinical Staging for HIV and AIDS

**S5 Table. The median time to death amongst deaths in the clinic and the median time to defaulting amongst all clinic defaulters.**

| **Dead or defaulted** | **Median time (IOR)** |
| --- | --- |
| Died | 4.73 (0.99-25.25 |
| Defaulted | 9.66 (1.31-35.60) |

**S6 Table. Default rates by 6 age group categorisation.**

|  | **Default rates** |
| --- | --- |
| Adult | 0.0595 |
| Young adult | 0.1006 |
| Older teenager | 0.0948 |
| Younger teenager | 0.0657 |
| Older child | 0.0591 |
| Young child | 0.1223 |
| Overall | 0.0654 |

**S7 Table. Log-rank test comparing default rates by 6 age group categorisation.**

|  | **Observed** | **Expected** | **p-value** |
| --- | --- | --- | --- |
| Adult | 4212 | 4540.34 | <0.001 |
| Young adult | 496 | 352.00 |  |
| Older teenager | 155 | 116.23 |  |
| Younger teenager | 173 | 180.38 |  |
| Older child | 162 | 186.59 |  |
| Young child | 451 | 273.46 |  |
